# 2020年中国慢性髓性白血病患者酪氨酸激酶抑制剂治疗状况调研

**DOI:** 10.3760/cma.j.issn.0253-2727.2021.07.002

**Published:** 2021-07

**Authors:** 慧芳 王, 龑莉 张, 晓力 刘, 焕玲 朱, 蓉 梁, 兵城 刘, 励 周, 力 孟, 纬明 黎, 倩 江

**Affiliations:** 1 北京大学人民医院、北京大学血液病研究所、国家血液系统疾病临床医学研究中心，北京 100044 Peking University People's Hospital, Peking University Institute of Hematology, National Clinical Research Center for Hematologic Disease, Beijing 100044, China; 2 河南省肿瘤医院血液科，郑州 450008 Department of Hematology, Affiliated Cancer Hospital of Zhengzhou University, Henan Provincial Tumor Hospital, Zhengzhou 450008, China; 3 南方医科大学南方医院血液科，广州 510515 Department of Hematology, Nanfang Hospital, Southern Medical University, Guangzhou 510515, China; 4 四川大学华西医院血液科，成都 610041 Department of Hematology, West China Hospital, Sichuan University, Chengdu 610041, China; 5 空军军医大学西京医院血液科，西安 510370 Department of Hematology, Xijing Hospital, Air Force Medical University, Xi'an 510370, China; 6 中国医学科学院血液病医院（中国医学科学院血液学研究所），天津 300020 Institute of Hematology, Chinese Academy of Medical Science, Tianjin 300020, China; 7 上海交通大学附属瑞金医院血液科，上海 200025 Shanghai Institute of Hematology, State Key Laboratory of Medical Genomics, National Research Center for Translational Medicine at Shanghai, Ruijin Hospital Affiliated to Shanghai Jiao Tong University School of Medicine, Shanghai 200025, China; 8 华中科技大学同济医学院附属同济医院血液科，武汉 430030 Department of Hematology, Tongji Hospital, Tongji Medical College, Huazhong University of Science and Technology, Wuhan 430030, China; 9 华中科技大学同济医学院附属协和医院血液科，武汉 430022 Department of Hematology, Union Hospital, Tongji Medical College, Huazhong University of Science and Technology, Wuhan 430022, China

**Keywords:** 白血病，髓性，慢性, 社会人口学特征, 酪氨酸激酶抑制剂, 药物转换, 治疗反应, 问卷调查, Leukemia, myeloid, chronic, Socio-demographic characteristics, Tyrosine kinase inhibitor, Drug switch, Treatment response, Questionnaires

## Abstract

**目的:**

调查中国慢性髓性白血病（CML）患者的治疗现状。

**方法:**

横断面研究，2020年4月末至5月中旬，以填写调研问卷的形式在全国范围内调研CML患者，分析酪氨酸激酶抑制剂（TKI）一线选择、目前用药、药物转换和获得主要分子学反应（MMR）的比例及其影响因素。

**结果:**

2933份来自全国31个省市自治区CML受访者的问卷可供分析，男性1683例（57.4％），中位年龄38（16～87）岁。一线选择：伊马替尼2481例（84.6％），原创性新药（原研药）1803例（61.5％）。填写问卷时用药：伊马替尼1765例（60.2％），原研药1791例（61.1％）。共1185例（40.4％）受访者曾经历TKI药物转换。1944例初发慢性期受访者TKI中位治疗45（3～227）个月，1417例（72.9％）获得≥MMR的疗效。多因素分析显示，城镇户籍（*OR*＝0.6，95％*CI* 0.5～0.8，*P*<0.001）、≥大学学历（*OR*＝0.5，95％*CI* 0.4～0.7，*P*<0.001）和进展期（*OR*＝0.5，95％*CI* 0.3～0.8，*P*＝0.001）受访者更少首选仿制TKI，而来自中部地区受访者比东部地区更多首选国产仿制TKI（*OR*＝1.7，95％*CI* 1.4～2.0，*P*<0.001）。进展期受访者更多首选二代TKI（*OR*＝5.4，95％*CI* 3.6～8.2，*P*<0.001），≥60岁受访者更少首选二代TKI（*OR*＝0.4，95％*CI* 0.2～0.7，*P*＝0.002）。诊断时处于进展期（*OR*＝2.2，95％*CI* 1.6～3.2，*P*<0.001）、首选伊马替尼（*OR*＝2.0，95％*CI* 1.6～2.6，*P*<0.001）、首选国产仿制药（*OR*＝1.3，95％*CI* 1.1～1.6，*P*＝0.002）、诊断距开始TKI治疗的时间更长（*OR*＝1.2，95％*CI* 1.1～1.2，*P*<0.001）和服用TKI的时间更长（*OR*＝1.1，95％*CI* 1.0～1.1，*P*<0.001）与药物转换比例增高显著相关。城镇户籍（*OR*＝0.7，95％*CI* 0.6～0.8，*P*<0.001）、获≥MMR（*OR*＝0.6，95％*CI* 0.5～0.8，*P*<0.001）和疗效未知（*OR*＝0.7，95％*CI* 0.6～0.9，*P*＝0.003）与药物转换比例低显著相关。女性（*OR*＝1.4，95％*CI* 1.1～1.7，*P*＝0.003）、城镇户籍（*OR*＝1.6，95％*CI* 1.3～2.0，*P*<0.001）、初始服用伊马替尼（*OR*＝1.4，95％*CI* 1.1～1.9，*P*＝0.016）和TKI治疗时间更长（*OR*＝1.2，95％*CI* 1.2～1.3，*P*<0.001）与获得≥MMR显著相关，而年龄≥60岁（*OR*＝0.7，95％*CI* 0.4～1.0，*P*＝0.047）和药物转换（*OR*＝0.6，95％*CI* 0.5～0.7，*P*<0.001）与未获得MMR显著相关。

**结论:**

截至2020年，中国CML患者中大多数首选并持续服用伊马替尼，半数以上服用原研药。社会人口学特征和疾病分期影响了患者的TKI选择、药物转换和治疗反应。

## Abstract (en)

**Objective:**

To investigate the current status of treatment choice and responses in patients with chronic myeloid leukemia (CML) in China.

**Methods:**

From the end of April to mid-May in 2020, a cross-sectional survey, by filling out a survey questionnaire, was conducted to explore the first-line choice of tyrosine kinase inhibitors (TKI), current medications, drug switch and major molecular responses (MMR) as well as the variables associated with them in patients in China.

**Results:**

Data of 2933 respondents with CML from 31 provinces, municipalities, and autonomous regions across the country were included in this study. 1683 respondents (57.4％) were males. Median age was 38（16–87）years old. 2481 respondents (84.6％) received imatinib as first-line TKI; 1803（61.5％）, the original new drug (branded drug). When completing the questionnaire, 1765 respondents（60.2％）were receiving imatinib; 1791（61.1％）, branded drug. 1185 respondents（40.4％）had experienced TKI switch. With a median follow-up of 45（3–227）months, 1417 of 1944（72.9％）respondents with newly diagnosed CML in the chronic phase achieved MMR. Multivariate analysis showed that the respondents with urban household registration（*OR*＝0.6, 95％*CI* 0.5–0.8, *P*<0.001）, ≥ bachelor degree（*OR*＝0.5, 95％*CI* 0.4–0.7, *P*<0.001）, and in the advanced phase at diagnosis（*OR*＝0.5, 95％*CI* 0.3–0.8, *P*＝0.001）less preferred Chinese generic TKI, while the respondents from the central region in China more preferred Chinese generic TKI more than those from the eastern region（*OR*＝1.7, 95％*CI* 1.4–2.0, *P*<0.001）. Moreover, the respondents in the advanced phase at diagnosis more preferred second-generation TKI（*OR*＝5.4, 95％*CI* 3.6–8.2, *P*<0.001）; those ≥60 years old, less preferred second-generation TKI（*OR*＝0.4, 95％*CI* 0.2–0.7, *P*＝0.002）. Being in the advanced phase at diagnosis（*OR*＝2.2, 95％*CI* 1.6–3.2, *P*<0.001）, first-line choice of imatinib（*OR*＝2.0, 95％*CI* 1.6–2.6, *P*<0.001）or Chinese generic drugs（*OR*＝1.3, 95％*CI* 1.1–1.6, *P*＝0.002）, longer interval from diagnose of CML to starting TKI treatment（*OR*＝1.2, 95％*CI* 1.1–1.2, *P*<0.001）and longer duration of TKI therapy（*OR*＝1.1, 95％ *CI* 1.0–1.1, *P*<0.001）were significantly associated with TKI switch; urban household registration（*OR*＝0.7, 95％*CI* 0.6–0.8, *P*<0.001）, ≥MMR（*OR*＝0.6, 95％*CI* 0.5–0.8, *P*<0.001）and unknown response（*OR*＝0.7, 95％*CI* 0.6–0.9, *P*＝0.003）, no TKI switch. Female sex（*OR*＝1.4, 95％*CI* 1.1–1.7, *P*＝0.003）, urban household registration（*OR*＝1.6, 95％*CI* 1.3–2.0, *P*<0.001）, front-line imatinib therapy（*OR*＝1.4, 95％ *CI* 1.1–1.9, *P*＝0.016）and longer duration of TKI treatment（*OR*＝1.2, 95％*CI* 1.2–1.3, *P*<0.001）were significantly associated with achieving a MMR or better response; age ≥ 60 years old（*OR*＝0.7, 95％*CI* 0.4–1.0, *P*＝0.047）and TKI switch（*OR*＝0.6, 95％ *CI* 0.5–0.7, *P*<0.001）, achieving no MMR.

**Conclusion:**

By 2020, the majority of Chinese CML patients received imatinib as the fist-line TKI therapy and continue to take it. More than half of TKIs were branded drugs. Socio-demographic characteristics and clinical variables affect their TKI choice, drug switch, and treatment response.

2000年后，第一代酪氨酸激酶抑制剂（TKI）伊马替尼的问世改变了慢性髓性白血病（CML）的治疗模式，显著改善了CML患者的生存，使CML成为可以长期生存的癌症。之后又有多种二代和三代TKI先后问世。随着原创性新药（原研药）TKI化合物专利过期，2013年10月中国国产仿制药（指与原研药在剂量、安全性、效力、质量、作用以及适应证上相同的药物）伊马替尼及达沙替尼先后上市，2018年11月，国产原研药氟马替尼上市，中国CML患者面临更多TKI选择。长期以来，TKI价格昂贵、治疗周期长、未进入医保目录等因素严重影响了中国CML患者TKI治疗的可及性。国外研究表明，CML患者的TKI选择、治疗依从性、治疗反应和结局与人口学特征、社会经济状况相关[1]–[4]。我们曾报道在2014年进行的一项中国CML患者TKI用药和治疗状况的调研[5]，当时绝大多数TKI未被国家和地方医保政策覆盖，结果显示84％的患者服用伊马替尼，81％的患者服用原研药。2017年，伊马替尼和达沙替尼进入全国医保目录。2018年，尼洛替尼进入全国医保目录。近年来，在国家和地方医保政策显著改善的同时，TKI价格逐渐降低，上述因素是否改变了中国CML患者的用药格局和治疗结果尚无报道。本研究旨在通过横断面调研，了解中国CML患者的TKI选择、药物转换和治疗反应，结合社会人口学特征，分析相关影响因素。

## 病例与方法

1. 研究设计：本研究为大型横断面研究，采用患者报告的形式，数据取自于2020年4月末至5月中旬开展的一项“疫情期间慢性髓性白血病患者心理状况调研”。该调研问卷通过问卷网制作，并通过微信平台向中国CML患者发放电子调查问卷。本研究获得北京大学人民医院伦理委员会的批准。调研对象为≥16岁的CML患者。

2. 调研问卷：问卷包括3个部分，共计76个问题。第一部分：受访者人口学特征（性别、年龄、户籍、居住地、婚姻状况、学历）、是否存在共存疾病，CML疾病特征（诊断时间、诊断时疾病分期），初始TKI治疗临床信息（开始TKI治疗距诊断时间、初始服用TKI种类、治疗时间、是否药物转换、药物转换种类、最新的治疗反应等）。第二部分包括疫情期间的患者行为和精神心理量表评估。第三部分是问卷有效性测评。本研究主要关注问卷的第一部分内容。

3. 统计学处理：患者人口学及疾病特征采用描述性统计分析，分类变量采用比例，连续性变量采用中位数，组间比较使用Person卡方检验、Mann-Whitney *U*和Wilcoxon检验。单因素分析*P*<0.2的变量纳入二元Logistic回归模型进行多因素分析。*P*<0.05为差异差异有统计学意义。采用SPSS 22.0软件进行统计分析。采用R-4.0.2软件制作桑基图。

## 结果

2020年4月28日至2020年5月12日，共收集来自中国除港、澳、台地区的31个省、自治区和直辖市的共3581份问卷，其中648份因重复填写（271份）、<16岁（71份）、初诊疾病分期未知（102份）、初始服用外国仿制药（200份）和尚未开始TKI治疗（4份）而被删除，最终，2933份有效问卷被纳入本研究进行分析。

1. 受访者特征：2933例可评估的受访者特征如表1所示。男性1683例（57.4％），诊断时中位年龄为38（16～87）岁，城镇户籍1744例（59.5％），已婚2337例（79.7％），≥大学学历1323例（45.1％）。中国东部地区1394例（47.5％），中部地区976例（33.3％），西部地区563例（19.2％）。2796例（95.3％）受访者诊断时处于慢性期，2627例（89.6％）受访者在诊断后6个月内开始TKI治疗，CML中位病程为49（1～325）个月，TKI中位治疗时间为47（1～228）个月。

**表1:** 2933例慢性髓性白血病（CML）受访者的特征

特征	总体^a^（2933例）	伊马替尼（2481例）	达沙替尼（92例）	尼洛替尼（337例）	*P*值
男性［例（％）］	1683（57.4）	1412（56.9）	53（57.6）	205（60.8）	0.394
诊断时年龄［例（％）］					0.003
<40岁	1618（55.2）	1339（54.0）	49（53.3）	218（64.7）	
40～60岁	1106（37.7）	952（38.4）	37（40.2）	106（31.5）	
≥60岁	209（7.1）	190（7.7）	6（6.5）	13（3.9）	
诊断时年龄［岁，*M*（范围）］	38（16～87）	38（16～87）	39（16～64）	35（17～73）	0.004
城镇户籍［例（％）］	1744（59.5）	1482（59.7）	44（47.8）	204（60.5）	0.067
婚姻状况［例（％）］					0.005
已婚	2337（79.7）	1994（80.5）	73（79.3）	250（74.2）	
未婚	392（13.4）	308（12.4）	14（15.2）	67（19.9）	
离异或丧偶	204（7.0）	179（7.2）	5（5.4）	20（5.9）	
学历［例（％）］					<0.001
≤初中	917（31.3）	772（31.1）	43（46.7）	94（27.9）	
高中	693（23.6）	611（24.6）	16（17.4）	63（18.7）	
≥大学	1323（45.1）	1098（44.3）	33（35.9）	180（53.4）	
居住地区［例（％）］					0.158
东部地区	1394（47.5）	1181（47.6）	35（38.0）	169（50.1）	
中部地区	976（33.3）	825（33.3）	40（43.5）	100（29.7）	
西部地区	563（19.2）	475（19.1）	17（18.5）	68（20.2）	
有合并症［例（％）］	683（23.3）	589（23.7）	21（22.8）	70（20.8）	0.478
有心血管合并症［例（％）］	302（10.3）	270（10.9）	8（8.7）	24（7.1）	0.091
诊断分期［例（％）］					<0.001
慢性期	2796（95.3）	2403（96.9）	77（83.7）	294（87.2）	
加速期	118（4.0）	65（2.6）	11（12.0）	41（12.2）	
急变期	19（0.6）	13（0.5）	4（4.3）	2（0.6）	
诊断距开始TKI治疗≤6个月［例（％）］	2627（89.6）	2209（89.0）	79（85.9）	319（94.7）	0.003
总病程［月，*M*（范围）］	49（1～325）	52（1～325）	38（2～249）	37（1～264）	<0.001
TKI治疗时间［月，*M*（范围）］	47（1～228）	50（1～228）	36（2～146）	35（1～133）	<0.001
一线TKI治疗［例（％）］					<0.001
原研TKI	1803（61.5）	1426（57.5）	28（30.4）	337（100.0）	
仿制TKI	1130（38.5）	1055（42.5）	64（69.6）	0	
药物转换比例［例（％）］	1185（40.4）	1065（42.9）	29（31.5）	81（24.0）	<0.001
药物转换类型［例（％）］					<0.001
原研和仿制药之间转换^b^	156（5.3）	151（6.1）	1（1.1）	4（1.2）	
伊马替尼和二代TKI之间转换^b^	861（29.4）	785（31.6）	18（19.6）	51（15.1）	
二代TKI之间转换^b^	151（5.1）	102（4.1）	12（13.0）	33（9.8）	
目前治疗［例（％）］					<0.001
伊马替尼	1765（60.2）	1718（69.2）	9（9.8）	32（9.5）	
二代TKI	1037（35.4）	649（26.2）	81（88.0）	291（86.4）	
三代TKI	118（4.0）	103（4.2）	2（2.2）	12（3.6）	
化疗、移植、原料药	13（0.4）	1.1（0.4）	0（0.0）	2（0.6）	
治疗线数［例（％）］					0.067
一线	2119（72.2）	1774（71.5）	65（70.7）	265（78.6）	
二线	622（21.2）	537（21.6）	20（21.7）	59（17.5）	
≥三线	192（6.5）	170（6.9）	7（7.6）	13（3.9）	
治疗效果［例（％）］					<0.001
未达CCyR	284（9.7）	222（8.9）	19（20.7）	38（11.3）	
CCyR	373（12.7）	300（12.1）	11（12.0）	59（17.5）	
MMR及更佳	1661（56.0）	1443（58.2）	34（37.0）	173（51.3）	
未知	615（21.0）	516（20.8）	28（30.4）	67（19.9）	

注：^a^年龄大于16岁且确诊超过3个月的CML患者；^b^包括1次及以上的药物转换。CCyR：完全细胞遗传学反应；MMR：主要分子学反应；TKI：酪氨酸激酶抑制剂

2. 一线TKI选择：不同年代患者一线TKI选择如图1所示。2019年开始，一线选择二代TKI的比例明显增加。全部受访者中，一线TKI选择伊马替尼2481例（84.6％），尼洛替尼337例（11.5％），达沙替尼92例（3.1％），其中原研药1803例（61.5％），国产仿制药1130例（38.5％）。填写问卷时，服用伊马替尼受访者1765例（60.2％），原研药1791例（61.1％）。其中，2119例（72.2％）受访者仍维持服用初始选择的TKI（不包括同种TKI之间的转换）。2013年10月以前诊断和2013年10月以后诊断的CML受访者初始和目前用药如表2所示。

2013年10月以前，由于可选择TKI有限，一线TKI中82.8％为原研药伊马替尼。2013年10月以后，国产伊马替尼和达沙替尼仿制药面市，患者有了多种选择，因此，我们分析了在2013年10月后确诊并接受TKI一线治疗的2113例受访者的初始用药选择，其中服用伊马替尼、尼洛替尼和达沙替尼者分别为1723例（82.2％）、302例（14.3％）、70例（3.3％），共计2095例。服用拉多替尼（临床试验）、氟马替尼者分别为12例（0.6％）和6例（3％）。服用原研药1083例（51.3％）。填写问卷时，伊马替尼、尼洛替尼和达沙替尼的中位治疗时间分别为35（1～79）、31（1～89）和30（2～78）个月，分别有1275例（74.0％）、246例（81.5％）和48例（68.6％）受访者仍维持初始一线TKI治疗。

**图1:**
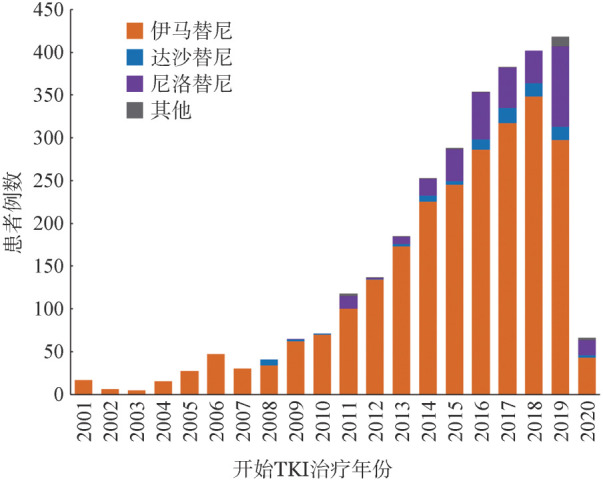
受访者开始酪氨酸激酶抑制剂（TKI）治疗的时间和一线TKI种类

**表2:** 受访者的酪氨酸激酶抑制剂（TKI）选择（％）

TKI种类	总体		2013年以前		2013年以后	
	初始	目前	初始	目前	初始	目前
格列卫	48.7	30.5	82.8	45.0	35.4	24.8
昕维	23.0	19.2	6.3	7.4	29.4	23.7
格尼可	11.6	8.2	3.2	5.4	14.8	9.3
诺利宁	1.4	0.8	0.2	0.4	1.9	1.0
施达赛	0.9	3.4	1.5	5.4	0.7	2.7
依尼舒	2.2	8.7	1.1	7.0	2.6	9.3
达希纳	11.5	22.2	4.3	18.0	14.3	23.8
其他	0.8	7.1	0.6	9.8	0.9	6.3

注：其他包括拉多替尼、耐克替尼、普纳替尼、氟马替尼和外国仿制药

2013年10月以后确诊的2113例CML受访者用于分析一线TKI选择的影响因素，纳入因素包括性别、诊断时年龄、户籍、婚姻状况、学历、地区、是否合并疾病、诊断分期等。多因素分析结果显示，城镇户籍（*OR*＝0.6，95％*CI* 0.5～0.8，*P*<0.001）、≥大学学历（*OR*＝0.5，95％*CI* 0.4～0.7，*P*<0.001）和诊断时处于进展期（*OR*＝0.5，95％*CI* 0.3～0.8，*P*＝0.001）患者更少首选仿制TKI，而来自中部地区的受访者比东部地区更多首选仿制TKI（*OR*＝1.7，95％*CI* 1.4～2.0，*P*<0.001）。诊断时处于进展期受访者更多首选二代TKI（*OR*＝5.4，95％*CI* 3.6～8.2，*P*<0.001），≥60岁受访者更少首选二代TKI（*OR*＝0.4，95％*CI* 0.2～0.7，*P*＝0.002）（表3）。

**表3:** 影响慢性髓性白血病（CML）患者TKI选择和疗效的多因素分析

影响因素	一线TKI选择^a^						药物转换^b^			达MMR^c^		
	仿制TKI			二代TKI			*OR*	95％*CI*	*P*值	*OR*	95％*CI*	*P*值
	*OR*	95％*CI*	*P*值	*OR*	95％*CI*	*P*值						
女性										1.4	1.1～1.7	0.003
诊断年龄						0.005						0.089
<40岁（参考组）												
40～60岁				0.8	0.6～1.0	0.085				1.0	0.8～1.3	0.737
≥60岁				0.4	0.2～0.7	0.002				0.7	0.4～1.0	0.047
婚姻						0.100						
未婚（参考组）												
已婚				0.8	0.6～1.0	0.093						
离异或丧偶				0.5	0.3～1.0	0.052						
城镇户籍	0.6	0.5～0.8	<0.001				0.7	0.6～0.8	<0.001	1.6	1.3～2.0	<0.001
学历			<0.001									
≤初中（参考组）												
高中	1.0	0.8～1.3	0.888	0.7	0.5～1.0	0.078						
≥大学	0.5	0.4～0.7	<0.001	1.1	0.8～1.4	0.476						
地区			<0.001									
东部地区（参考组）												
中部地区	1.7	1.4～2.0	<0.001									
西部地区	1.2	0.9～1.5	0.142									
有合并症										1.3	1.0～1.7	0.071
诊断时处于进展期	0.5	0.3～0.8	0.001	5.4	3.6～8.2	<0.001	2.2	1.6～3.2	<0.001			
初始服用伊马替尼							2.0	1.6～2.6	<0.001	1.4	1.1～1.9	0.016
初始服用国产TKI仿制药							1.3	1.1～1.6	0.002			
诊断距开始TKI治疗时间（年）							1.2	1.1～1.2	<0.001			
TKI治疗时间（年）							1.1	1.0～1.1	<0.001	1.2	1.2～1.3	<0.001
药物转换										0.6	0.5～0.7	<0.001
治疗反应									<0.001			
<MMR（参考组）												
≥MMR							0.6	0.5～0.8	<0.001			
未知							0.7	0.6～0.9	0.003			

注：^a^纳入2013年10月以后确诊的2113例CML患者；^b^纳入研究的2933例CML患者；^c^纳入诊断距开始TKI治疗时间≤6个月、TKI治疗时间≥3个月且目前正在接受TKI治疗的1944例慢性期CML患者；MMR：主要分子学反应；TKI：酪氨酸激酶抑制剂

3. 药物转换：在全部2933例受访者中，1185例（40.4％）受访者经历药物转换（包括同种TKI之间的转换，如原研药和仿制药之间的转换和不同品牌之间的转换）。这些受访者中，115例（3.9％）是同代原研药换仿制药，44例（1.5％）是同代仿制药换原研药，861例（29.4％）是伊马替尼和二代TKI之间互换，151例（5.1％）是二代TKI之间互换。814例（27.8％）患者经历了2种TKI转换，192例（6.5％）患者经历了3种TKI转换，27例（0.9％）经历了4种TKI转换（图2）。

**图2:**
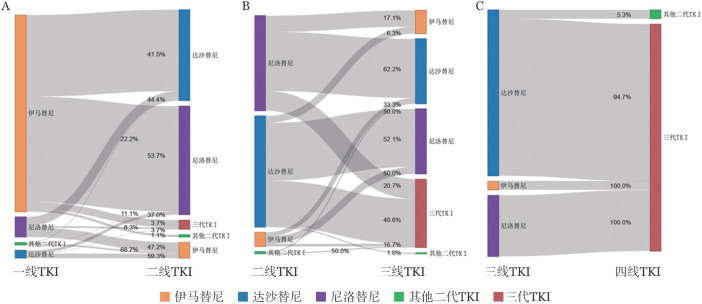
受访者酪氨酸激酶抑制剂（TKI）药物转换 A：一线TKI（伊马替尼706例，达沙替尼27例，尼洛替尼72例，其他二代TKI 9例）转换为二线TKI；B：二线TKI（伊马替尼12例，达沙替尼96例，尼洛替尼82例，其他二代TKI 2例）转换为三线TKI；C：三线TKI（伊马替尼1例，达沙替尼19例，尼洛替尼7例）转换为四线TKI。其他二线TKI包括拉多替尼和氟马替尼；三代TKI包括普纳替尼和耐克替尼

纳入社会人口学特征、合并疾病、诊断分期、初始服用TKI种类（伊马替尼/二代TKI，原研/仿制）、诊断距开始TKI治疗时间、TKI治疗时间和治疗反应等因素分析药物转换的影响因素，多因素分析结果显示：诊断时处于进展期（*OR*＝2.2，95％*CI* 1.6～3.2，*P*<0.001）、首选伊马替尼（*OR*＝2.0，95％ *CI* 1.6～2.6，*P*<0.001）、首选国产仿制药（*OR*＝1.3，95％*CI* 1.1～1.6，*P*＝0.002）、诊断距开始TKI治疗的时间更长（*OR*＝1.2，95％*CI* 1.1～1.2，*P*<0.001）和服用TKI的时间更长（*OR*＝1.1，95％*CI* 1.0～1.1，*P*<0.001）与药物转换比例增高显著相关，而城镇户籍（*OR*＝0.7，95％*CI* 0.6～0.8，*P*<0.001）、获得≥主要分子学反应（MMR）（*OR*＝0.6，95％*CI* 0.5～0.8，*P*<0.001）和疗效未知（*OR*＝0.7，95％*CI* 0.6～0.9，*P*＝0.003）与更少药物转换显著相关（表3）。

4. 治疗反应：在2933例受访者中，2318例（79.0％）受访者报告了自己填写问卷时的治疗反应：373例（16.1％）报告Ph染色体转阴，即获得完全细胞遗传学反应（CCyR），1661例（71.7％）获得≥MMR（其中964例报告基因转阴）。

在总人群中报告了分子学反应、诊断距开始TKI治疗时间≤6个月、TKI服用时间≥3个月且目前正在接受TKI治疗的1944例慢性期CML受访者用于分析获得MMR的影响因素，其中1417例（72.9％）报告获得≥MMR的分子学反应。纳入社会人口学和临床特征以及是否药物转换等因素分析分子学反应的影响因素，多因素分析结果显示，女性（*OR*＝1.4，95％*CI* 1.1～1.7，*P*＝0.003）、城镇户籍（*OR*＝1.6，95％*CI* 1.3～2.0，*P*<0.001）、首选伊马替尼（*OR*＝1.4，95％*CI* 1.1～1.9，*P*＝0.016）和TKI治疗时间更长（*OR*＝1.2，95％ *CI* 1.2～1.3，*P*<0.001）与获得≥MMR显著相关，而年龄≥60岁（*OR*＝0.7，95％*CI* 0.4～1.0，*P*＝0.047）和经历药物转换（*OR*＝0.6，95％*CI* 0.5～0.7，*P*<0.001）与未获得MMR显著相关（表3）。

## 讨论

本研究是2020年在中国CML患者中进行的大型调研。调研发现，中国CML患者的一线TKI选择以伊马替尼为主，TKI中位治疗近4年后，72.2％的受访者仍维持初始TKI治疗，40.4％的受访者经历药物转换，71.7％达MMR。社会人口学特征（如性别、年龄、户籍、学历、居住地区等）和临床特征（共存疾病、疾病分期等）影响了患者的用药选择和治疗反应。

近6年来，随着国产仿制TKI上市、医保政策改善、TKI降价及人们生活水平提高，TKI可及性更高，这些变化导致了TKI结构的变迁。2014年的调研显示，94％受访者初诊时处于慢性期，78％在诊断1年内开始服用TKI，84％服用伊马替尼，81％服用原研药[5]。与2014年的调研结果相比，本研究受访者在初诊时处于慢性期的比例相同，而诊断至开始接受TKI治疗的时间更短，体现了目前TKI的可及性更高。同时，TKI的选择也发生了改变，本次调研时更多的中国CML受访者服用国产仿制TKI（37％对7％）和二代TKI（35.4％对16％）。在各种TKI均可及且国产TKI的疗效和安全性已被认可的当下，医师可以根据CML患者的人口学特征和疾病特征，制定更加个体化的TKI治疗方案[6]–[9]。

不难理解，由于中国城乡和地区经济发展的差异，多数城镇户籍、高学历和东部地区的受访者有更高的经济能力支付昂贵的原研药和二代TKI治疗费用，这一结果与一项来自瑞士的研究结果一致[3]。二代TKI在中高危慢性期和进展期CML患者中疗效优于伊马替尼，可以使患者更快、更深地获得细胞遗传学和分子学反应，降低疾病进展率[10]–[13]，因此，可以理解本研究中初诊时处于进展期患者一线更多选择二代TKI，也体现了医患对TKI选择的认知符合国内外CML管理指南的推荐和专家共识[14]–[15]。

本调研受访者中，药物转换比例为40.4％，其中5.3％为同代原研和仿制TKI之间的转换。由于本调研设计中未涉及受访者药物转换的原因，但根据既往报道，CML药物转换的原因依次为耐药、不耐受和个人原因[16]–[17]。推测，同代原研和仿制TKI之间的转换主要受当地医保政策变化的影响，也不除外医患对仿制TKI有效性和安全性的信赖或患者个人经济收入水平和药品支付能力的变化[6]–[9]。在TKI种类多样化的当下，CML患者的用药选择和换药空间更大，尤其是耐药后有更多的药物选择。

本研究发现，城镇户籍CML患者获得MMR比例更高，分析可能与城镇户籍受访者的社会经济地位更高和医疗资源更丰富有关。与既往报道不同，本研究发现一线选择伊马替尼而非二代TKI的受访者更多获得MMR，可能与真实世界中更多中/高危慢性期患者选择二代TKI有关。国外研究报道，年龄不影响CML患者TKI治疗反应的获得[18]–[19]，而本调研结果显示年龄≥60岁不利于获得MMR，可能是因为老年CML患者共存疾病多、非血液学不良反应发生率高、服用减量TKI的比例高，导致老年CML患者深度分子学反应获得率低[20]–[21]。与欧洲SIMPLICITY研究结果[17]相同，本研究也发现药物转换与未获得MMR显著相关。21.0％的受访者不清楚自己的治疗反应，反映了部分患者对疾病认知度低或重视程度不足，或对化验单不会解读。提示需要更多开展CML患者教育，以提高患者对疾病的认知和自我管理能力，促进与医师的配合，最终改善治疗结局。

本研究存在以下缺陷：①通过发放电子问卷进行调研，某些老年或文化程度较低患者未能纳入本研究，存在受访者选择偏倚；②由于调研形式为患者自我报道的限制，本研究没有在问卷中设计询问患者的疾病危险度（如Sokal评分和ELTS评分等），因此未对患者的用药选择和治疗反应进行按疾病危险度分层的分析比较；③受访者对疾病诊断、治疗用药和治疗反应的回忆信息可能不准确；④考虑到隐私安全，受访者和其家庭的收入信息未被列入收集范围，而这些可能是影响受访者TKI选择的重要因素；⑤不同实验室采用的检测方法可能存在差异，导致对TKI反应的评估难以精确到MMR以上的深层分子学反应。

总之，本研究分析了当今中国CML患者的TKI用药和治疗反应状态。医保政策的完善和TKI药价的降低，显著改善了CML患者TKI的可及性。截至2020年，中国CML患者仍以伊马替尼为主要选择，所有TKI中原研药占半数。人口学特征和疾病分期影响了CML患者的TKI选择、药物转换和治疗反应。

## References

[1] Smith AG, Painter D, Howell DA (2014). Determinants of survival in patients with chronic myeloid leukaemia treated in the new era of oral therapy: findings from a UK population-based patient cohort[J]. BMJ Open.

[2] Perry AM, Brunner AM, Zou T (2017). Association between insurance status at diagnosis and overall survival in chronic myeloid leukemia: A population-based study[J]. Cancer.

[3] Larfors G, Sandin F, Richter J (2017). The impact of socio-economic factors on treatment choice and mortality in chronic myeloid leukaemia[J]. Eur J Haematol.

[4] Ylescas-Soria J, de la Torre-Lujan AH, Herrera LA (2019). Prognostic factors for overall survival in patients with chronic myeloid leukemia treated with imatinib at the National Cancer Institute - Mexico, from 2000 to 2016[J]. Cancer Med.

[5] 江 倩, 刘 正琛, 张 颂昕 (2016). 中国慢性髓性白血病患者酪氨酸激酶抑制剂治疗现状的调研: 从患者的角度[J]. 中华血液学杂志.

[6] Dou X, Qin Y, Lai Y (2020). Comparable Efficacy and Safety of Generic Imatinib and Branded Imatinib in Patients With Newly Diagnosed Chronic Myeloid Leukemia With a Consideration of Socioeconomic Characteristics: A Retrospective Study From a Single Center[J]. Clin Lymphoma Myeloma Leuk.

[7] 石 红霞, 秦 亚溱, 赖 悦云 (2016). 国产与原研伊马替尼治疗初发慢性髓性白血病慢性期患者有效性和安全性比较的单中心、前瞻性队列研究[J]. 中华内科杂志.

[8] 窦 雪琳, 于 露, 秦 亚溱 (2019). 结合人口学特征比较国产和原研伊马替尼治疗慢性髓性白血病慢性期患者的有效性和安全性[J]. 中华血液学杂志.

[9] 赵 婷, 于 露, 秦 亚溱 (2019). 原研伊马替尼或尼洛替尼转换国产伊马替尼治疗慢性髓性白血病慢性期患者有效性、安全性和生活质量研究[J]. 中华血液学杂志.

[10] Wang J, Shen ZX, Saglio G (2015). Phase 3 study of nilotinib vs imatinib in Chinese patients with newly diagnosed chronic myeloid leukemia in chronic phase: ENESTchina[J]. Blood.

[11] Giles FJ, le Coutre PD, Pinilla-Ibarz J (2013). Nilotinib in imatinib-resistant or imatinib-intolerant patients with chronic myeloid leukemia in chronic phase: 48-month follow-up results of a phase II study[J]. Leukemia.

[12] Giles FJ, Kantarjian HM, le Coutre PD (2012). Nilotinib is effective in imatinib-resistant or -intolerant patients with chronic myeloid leukemia in blastic phase[J]. Leukemia.

[13] Cortes J, Kim DW, Raffoux E (2008). Efficacy and safety of dasatinib in imatinib-resistant or -intolerant patients with chronic myeloid leukemia in blast phase[J]. Leukemia.

[14] Hochhaus A, Baccarani M, Silver RT (2020). European LeukemiaNet 2020 recommendations for treating chronic myeloid leukemia[J]. Leukemia.

[15] Baccarani M, Deininger MW, Rosti G (2013). European LeukemiaNet recommendations for the management of chronic myeloid leukemia: 2013[J]. Blood.

[16] Breccia M, Olimpieri PP, Olimpieri O (2020). How many chronic myeloid leukemia patients who started a frontline second-generation tyrosine kinase inhibitor have to switch to a second-line treatment? A retrospective analysis from the monitoring registries of the italian medicines agency (AIFA)[J]. Cancer Med.

[17] Gambacorti-Passerini C, Chen C, Davis C (2021). Treatment patterns and clinical outcomes of tyrosine kinase inhibitors in chronic-phase CML in clinical practice: 3-year European SIMPLICITY data[J]. Eur J Haematol.

[18] Bělohlávková P, Voglová J, Radocha J (2015). Impact of age on the clinical response of patients with CML treated with imatinib[J]. Vnitr Lek.

[19] Lokesh KN, Pehalajani JK, Loknatha D (2020). CML in Elderly: Does Age Matter?[J]. Indian J Hematol Blood Transfus.

[20] 于 露, 江 倩 (2018). 中国服用酪氨酸激酶抑制剂的慢性髓性白血病慢性期患者共存疾病状况及其对患者报告结果的影响[J]. 中华血液学杂志.

[21] 彭 楠, 窦 雪琳, 于 露 (2021). 初发慢性髓性白血病慢性期不同年龄患者临床特征、治疗和结局[J]. 中华血液学杂志.

